# Diclofenac–hyaluronate conjugate (diclofenac etalhyaluronate) intra-articular injection for hip, ankle, shoulder, and elbow osteoarthritis: a randomized controlled trial

**DOI:** 10.1186/s12891-022-05328-3

**Published:** 2022-04-20

**Authors:** Toshikazu Kubo, Tsukasa Kumai, Hiroyasu Ikegami, Kazuyuki Kano, Megumi Nishii, Takayuki Seo

**Affiliations:** 1 Kyoto Interdisciplinary Research Institute, Nakagyo-ku, Kyoto, Japan; 2 Faculty of Sport Sciences, Waseda University, Tokorozawa, Saitama, Japan; 3 Department of Orthopaedics Surgery (Ohashi), School of Medicine, Toho University, Meguro-ku, Tokyo, Japan; 4 Clinical Development Department, Research & Development Division, Seikagaku Corporation, 1-6-1 Marunouchi, Chiyoda-ku, Tokyo, 100-0005 Japan

**Keywords:** Osteoarthritis, Shoulder, Elbow, Hip, Ankle, NSAIDs, Hyaluronan, Intra-articular injection, Diclofenac etalhyaluronate

## Abstract

**Background:**

To evaluate the efficacy and safety of intra-articular injection of diclofenac etalhyaluronate (DF-HA) in patients with osteoarthritis (OA) of the hip, ankle, shoulder, or elbow.

**Methods:**

In this randomized, placebo-controlled, double-blind study in Japan, Japanese patients aged ≥20 years diagnosed with OA of the hip, ankle, shoulder, or elbow were randomly assigned 1:1 to DF-HA 30 mg or placebo (citric acid-sodium citrate buffered solution). Subjects received three injections of the study drug in each joint cavity every 4 weeks and were assessed for 12 weeks after the first injection. The primary endpoint was the mean change from baseline in a diary-based 11-point numerical rating scale (NRS) for pain over 12 weeks, analyzed for each joint. Treatment-emergent adverse events were recorded, and morphological changes in each joint were evaluated radiographically.

**Results:**

The study drug (DF-HA vs placebo) was injected into 90, 60, 90, or 50 subjects with OA of the hip, ankle, shoulder, or elbow (46 vs 44, 30 vs 30, 45 vs 45, and 25 vs 25, respectively). The group differences in the mean change from baseline in the pain NRS over 12 weeks were − 0.81 (95% confidence interval: − 1.48 to − 0.13), − 0.07 (− 1.03 to 0.89), 0.15 (− 0.48 to 0.78), and 0.61 (− 0.41 to 1.62) for the hip, ankle, shoulder, and elbow joints, respectively, with statistically significant differences observed only in the hip joint. The change from baseline in the hip joint was greater with DF-HA than placebo at all time points from Weeks 1–12. No clinically significant adverse events or radiographic changes were observed.

**Conclusions:**

Intra-articularly administered DF-HA for hip OA produced a rapid response and was safe, with analgesia maintained for 12 weeks when administered every 4 weeks.

**Trial registration:**

JapicCTI-173,678 (First registered date: 21 August 2017).

**Supplementary Information:**

The online version contains supplementary material available at 10.1186/s12891-022-05328-3.

## Background

Osteoarthritis (OA) is a joint disease that often affects middle-aged and older adults, and it reduces their quality of life (QOL) by interfering with activities of daily living owing to pain, swelling, and deformity [1, 2]. OA may occur in joints throughout the body, including the knee, hip, ankle, shoulder, and elbow joints. Drug therapy for OA is an important conservative treatment and is selected according to patients’ symptoms, concurrent diseases, and intentions [1–4]. Acetaminophen is the initial drug, followed by oral non-steroidal anti-inflammatory drugs (NSAIDs), which are the main drug therapy, but these are not recommended for long-term use because of systemic risks, such as gastrointestinal disorders, cardiovascular disorders, and renal dysfunction related to their use [5–8]. Topical NSAIDs are also used for patients with OA, but there is concern that these drugs cannot act completely, especially in the hip joint, which is far from the body surface [1]. Steroids and hyaluronic acid (HA) are used in the knee joint as intra-articular injection agents, but evidence is limited for other joints [1, 3]. A drug that can be used effectively and safely not only in the knee joint but also in other joints is needed.

Diclofenac etalhyaluronate (DF-HA), approved for treatment of knee and hip OA in Japan in March 2021, is a novel intra-articular injection agent of fermentation-derived HA (600,000 to 1,200,000 Da) chemically linked with diclofenac sodium (DF), an NSAID. DF-HA is expected to provide analgesia as well as improve joint function, as does conventional HA, but also to act more rapidly and persistently for up to 28 days owing to the anti-inflammatory effects of slow DF release into the joint cavity [9–11]. In addition, the systemic side effects of DF are expected to be lower than those of NSAIDs because of their local administration. In previous clinical studies in patients with knee OA, analgesia induced by DF-HA, which was administered intra-articularly every 4 weeks for a total of three doses, was maintained for 12 weeks from Week 1 [11, 12].

The aim of this study was to evaluate the efficacy and safety of intra-articular DF-HA administered every 4 weeks in joints with OA other than the knee, and especially to determine if DF-HA had similar efficacy and safety profiles in other joints affected by OA compared with the knee.

## Methods

### Study design and setting

This was a placebo-controlled, randomized, double-blind, parallel-group study conducted at 44 sites including university hospitals, clinics and general hospitals in Japan (JapicCTI-173,678, 21 August 2017). The study was conducted in accordance with the Declaration of Helsinki and Good Clinical Practice (GCP) guidelines after approval by the central institutional review board (IRB) or the respective IRBs. Written informed consent was obtained from all patients.

### Subjects

OA patients aged ≥20 years who had pain in the hip, ankle, shoulder, or elbow joint for at least 6 months, with a diary-based mean 11-point (0–10) numerical rating scale (NRS) for pain of 5–9 in the target joint during the screening period were eligible to participate in this study. In patients with OA involving multiple joints, only one joint was treated with the study drug and assessed as the target joint. In addition, the following inclusion criteria were specified for OA in each joint: 1. Hip OA diagnosed as primary OA or secondary OA owing to acetabular dysplasia and classified radiographically as early (mild articular wear) or advanced OA (advanced articular wear) according to the Japanese Orthopaedic Association stage classification of hip osteoarthritis [13]. 2. Ankle OA diagnosed as primary talocrural OA or secondary talocrural OA owing to trauma and classified radiographically as stage II (partial narrowing of the joint space) or III (partial disappearance of the joint space) varus ankle OA. 3. Shoulder OA diagnosed as primary glenohumeral OA or secondary glenohumeral OA owing to trauma or grade ≥ 4 rotator cuff tear according to the Hamada classification [14, 15], a radiographic classification of massive rotator cuff tear, and classified radiographically as Kellgren–Lawrence (KL) grade 2 or 3. 4. Elbow OA diagnosed as primary OA or secondary OA owing to trauma and classified radiographically as KL grade 2 or 3. Patients with serious diseases, such as cardiac, hepatic, or renal diseases, blood dyscrasia, or immunodeficiency, with a history of hypersensitivity to HA, DF-HA, or acetaminophen, who received any HA preparation in the target joint within the specified period, or with shoulder/elbow OA or hip/ankle OA with an NRS pain score ≥ 4 in any non-target joint in the upper or lower body were excluded from the study.

### Randomization and blinding

The study drugs were dynamically allocated by the minimization method using an interactive web response system incorporating a randomized algorithm, so that subjects were assigned to the DF-HA or placebo group in a 1:1 ratio for each joint [16]. The stratification factors were the site, cause of OA, stage of OA, baseline NRS for pain, sex, and age. DF-HA was more viscous than the placebo, requiring a higher pressure during injection; therefore, the difference in viscosity might have allowed the treating investigator to distinguish whether the study drug was DF-HA or placebo To maintain the study blinding, evaluations after the first injection until the end of the study were performed by an investigator who did not administer the study drug. A treating investigator administered the study drug to subjects after providing a written signed pledge not to disclose any information to the subjects or other staff that might affect the blindness of the study.

### Treatment method

Patients were screened from 1 week before randomization. Patients meeting the inclusion criteria received a total of three injections of either DF-HA (syringe filled with 3 mL of sodium citrate buffer including 30 mg DF-HA; Seikagaku Corporation, Tokyo, Japan) or placebo (syringe filled with 3 mL of sodium citrate buffer without DF-HA; Seikagaku Corporation) in the target joint cavity every 4 weeks (Weeks 0, 4, and 8). Because a 3-mL injection could be administered to all joints, the same dose regimen was selected as in previous studies of knee OA [11, 12]. The study drug was administered by an orthopedic surgeon according to the prescribed procedure for injection into each joint (Supplementary Table 1, Additional file 1). While ultrasound- or fluoroscopy-guided injection was recommended, blind injection was allowed for the ankle, shoulder, and elbow joints if administered by specialists (members of designated academic societies) who carried out the practices in daily medical practice. Oral NSAIDs, steroids, opioids, psychotherapeutic agents, and anesthetics, as well as intra-articular agents, including steroids, were prohibited during the study. Acetaminophen was used as rescue medication. During the study, existing physical therapy could be continued at the same frequency or intensity, but the initiation of new physical therapies was prohibited. Similarly, patients were instructed not to change the frequency or intensity of exercise in their daily activities. Removal of joint effusion from the target joint was prohibited unless the investigator judged it necessary before study drug injection.

### Evaluation methods

The primary outcome measure was the diary-based 11-point NRS for pain in the target joint, and the primary endpoint was the mean change from baseline in NRS for pain over 12 weeks after the first injection. Secondary endpoints comprised joint-specific endpoints for the hip, ankle, shoulder, and elbow joints over 12 weeks after the first injection using the Western Ontario and McMaster Universities Osteoarthritis 3.1 index (WOMAC) [17], Self-Administered Foot Evaluation Questionnaire (SAFE-Q) [18], Shoulder36 [19], and Patient-Rated Elbow Evaluation (Japanese Version) (PREE-J) [20], respectively. In addition, the proportion of responders, patient and physician global assessment scores (100-mm visual analog scale [VAS]), Medical Outcomes Study 36-Item Short Form Health Survey (SF-36) [21–23], EuroQol 5 Dimensions (EQ-5D) [24], joint range of motion, and acetaminophen consumption were assessed.

Safety was evaluated according to treatment-emergent adverse events (TEAE) discovered after the initial injection, which were reported in accordance with the definitions in Supplementary Table 2, Additional file 2. The relationship of TEAE to the study drug was assessed by a blinded investigator. TEAEs of special interest were TEAEs relating to the injection site, gastrointestinal disorders, cardiovascular disorders, renal dysfunction, hypersensitivity, and anaphylactic reaction. To confirm safety, the target joint was radiographed before the first injection of the study drug and at Week 12 or discontinuation. Joints were assessed by the investigator for morphological changes (osteophyte, joint space narrowing, osteosclerosis, and epiphyseal deformity) according to the study protocol (Supplementary Table 3, Additional file 3). In addition, manual joint examination, laboratory testing (hematology, blood biochemistry, and urinalysis), and vital signs measurement were performed, and the investigator determined whether any abnormal change in these four tests corresponded to a TEAE.

### Statistical analysis

According to the results of a phase 2 study in patients with knee OA [11], the between-group difference for each joint was assumed to be − 0.55, with a standard deviation of 2.00. To demonstrate that DF-HA was as effective for OA in each joint as for knee OA, the target sample size was calculated to be 45 subjects per group for each joint, with a 90% probability of the point estimate in the DF-HA group being above that in the placebo group. Given the small number of patients with ankle or elbow OA and the feasibility of study, the target sample sizes were calculated to be 30 and 20 patients per group, respectively, with 85 and 80% probabilities, respectively, of the point estimate in the DF-HA group being above that in the placebo group.

Efficacy was evaluated for each target joint in the full analysis set (FAS), which was a population comprising all subjects who had at least one post-treatment efficacy data value. The primary analysis was to compare the mean change from baseline in NRS for pain over 12 weeks after the first injection in each target joint between the DF-HA and placebo groups using a mixed model for repeated measures (MMRM) analysis. The fixed effects were treatment, time, treatment-by-time interaction, baseline NRS for pain, type of OA, stage of OA, age, and sex. The correlation between time points in subjects was assumed to be unstructured. The Kenward–Roger method was used to calculate the degrees of freedom. Secondary analyses comprised comparisons of the change from baseline in NRS for pain at each time point using the same MMRM analysis. In addition, the mean change from baseline over 12 weeks after the first injection was compared between the two groups for the following secondary endpoints using an MMRM analysis: joint-specific endpoints, and patient and physician global assessment scores. The proportion of responders, defined as patients with at least 30% improvement in NRS for pain from baseline, was calculated at each time point. In addition, the odds ratio was calculated using a generalized estimating equation (GEE). The explanatory variables in the model were treatment, time, treatment-by-time interaction, type of OA, stage of OA, diary-based baseline pain score in the target joint, age, and sex. An analysis of covariance was performed for the changes in SF-36 and EQ-5D from baseline to Week 12. The covariates were the baseline value of each outcome measure, type of OA, stage of OA, age, and sex. For joint range of motion and the mean daily consumption of acetaminophen, the change from baseline at each time point was summarized. Acetaminophen consumption was considered to be zero in subjects who did not take it. SAS software, version 9.4 (SAS Institute Japan Ltd., Tokyo, Japan) was used to perform the analyses.

Safety was evaluated in the safety set, a population consisting of all subjects who received at least one injection of the study drug. The incidences of TEAEs were tabulated for all joints combined and by joint. TEAEs were coded using the Medical Dictionary for Regulatory Activities (MedDRA) ver. 22.0. TEAEs of special interest other than those at the injection site were classified using the standardized MedDRA query. Other safety endpoints were also tabulated for all joints combined and by joint.

## Results

### Subjects

**Fig. 1:**
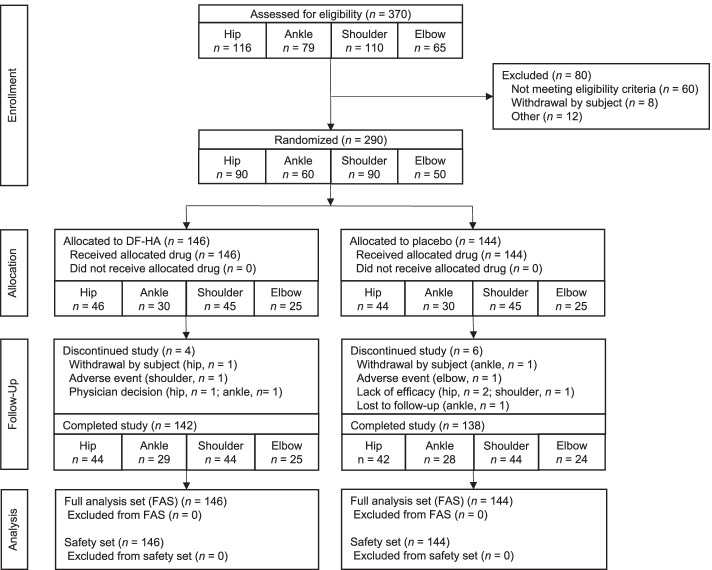
Study flow chart. DF-HA: diclofenac etalhyaluronate

From September 2017 to March 2019, 370 patients were screened for eligibility, and 290 patients were enrolled and randomized to receive DF-HA (hip in 46, ankle in 30, shoulder in 45, and elbow in 25) or placebo (hip in 44, ankle in 30, shoulder in 45, and elbow in 25) (Fig. 1). All 290 subjects were included in the FAS and safety set. The subjects’ demographics are presented by joint in Table 1. Regarding the classification of OA, the prevalence of secondary OA was higher in the hip joint than in the other joints, although hip OA in Japanese patients is characterized by a higher prevalence in female patients with acetabular dysplasia [25]. In addition, the subjects’ demographics and baseline values at each efficacy endpoint are presented by treatment group in Supplementary Table 4, Additional file 4.

**Table 1:** Patients’ demographic and baseline characteristics

Characteristic	Hip *N* = 90	Ankle *N* = 60	Shoulder *N* = 90	Elbow *N* = 50	Total *N* = 290
Age, years	59.9 ± 8.9	65.4 ± 11.6	70.2 ± 11.4	61.3 ± 13.0	64.5 ± 11.8
Sex					
Male	10 (11.1)	13 (21.7)	47 (52.2)	37 (74.0)	107 (36.9)
Female	80 (88.9)	47 (78.3)	43 (47.8)	13 (26.0)	183 (63.1)
BMI, kg/m^2^	23.43 ± 3.37	24.67 ± 3.54	24.03 ± 3.46	24.32 ± 3.47	24.03 ± 3.46
Duration of current joint pain, weeks	241.8 ± 255.7	188.3 ± 196.1	161.3 ± 186.4	358.5 ± 401.8	225.9 ± 266.0
Classification of OA					
Primary	18 (20.0)	42 (70.0)	69 (76.7)	43 (86.0)	172 (59.3)
Secondary	72 (80.0)	18 (30.0)	21 (23.3)	7 (14.0)	118 (40.7)
Stage of OA^a^, n (%)					
Stage A	27 (30.0)	19 (31.7)	53 (58.9)	22 (44.0)	121 (41.7)
Stage B	63 (70.0)	41 (68.3)	37 (41.1)	28 (56.0)	169 (58.3)
NRS for pain^b^, n (%)	6.94 ± 1.06	7.02 ± 1.06	6.63 ± 1.08	6.69 ± 0.93	6.82 ± 1.05

Data are presented as mean ± standard deviation or n (%)

*BMI* body mass index, *OA* osteoarthritis, *NRS* numerical rating scale

^a^Stage A: Kellgren–Lawrence (KL) grading score for hip OA stage (Early stage); ankle OA stage (Stage 1/ Stage 2); shoulder OA and elbow OA (Grade 2). Stage B: KL grading score for hip OA stage (Advanced stage); ankle OA stage (Stage 3); shoulder OA and elbow OA (Grade 3)

^b^Average of 7 days prior to Week 0 by the 0–10 numerical rating scale for pain intensity: 0 indicates no pain, and 10 indicates the worst pain

### Efficacy

**Fig. 2:**
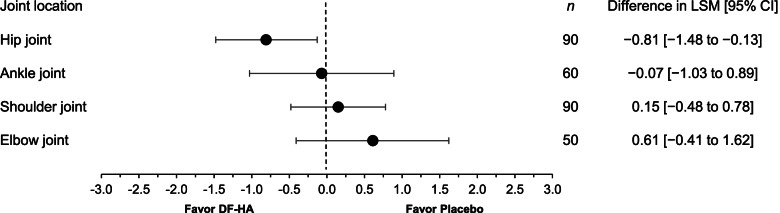
Mean change from baseline in NRS for pain over 12 weeks - full analysis set. Changes from baseline were estimated using a mixed model for repeated measures. LSM: least-squares means; CI: confidence interval; DF-HA: diclofenac etalhyaluronate

The mean changes from baseline in NRS for pain over 12 weeks after the first injection in the DF-HA and placebo groups were − 2.90 and − 2.10 for the hip joint, − 1.96 and − 1.89 for the ankle joint, − 1.84 and − 1.99 for the shoulder joint, and − 2.28 and − 2.89 for the elbow joint, respectively. The between-group difference (DF-HA group minus placebo group) and its 95% confidence interval (CI) was − 0.81 (− 1.48 to − 0.13), − 0.07 (− 1.03 to 0.89), 0.15 (− 0.48 to 0.78), and 0.61 (− 0.41 to 1.62) for the hip, ankle, shoulder, and elbow joint, respectively. A statistically significant difference was observed in the hip joint (Fig. 2). The change from baseline in the hip joint was greater in the DF-HA group than in the placebo group at all time points. In joints other than the hip joint, no improvement was observed compared with placebo (Supplementary Table 5, Additional file 5).

The mean changes from baseline in joint-specific endpoints over 12 weeks after the first injection of the study drug are presented in Table 2. In the WOMAC for the hip joint, a greater improvement in the DF-HA group than in the placebo group was observed for all subscores. In the SAFE-Q for the ankle joint, the Shoulder36 for the shoulder joint, and the PREE-J for the elbow joint, no clear improvement was observed in the DF-HA group; however, the changes in domains other than “pain and pain-related” in the ankle joint were greater in the DF-HA group than in the placebo group. The proportion of responders at each time point is detailed in Supplementary Table 6, Additional file 6. The proportions of responders at Week 12 in the DF-HA and placebo groups were 68.2 and 45.2% for the hip joint, 51.7 and 57.1% for the ankle joint, 56.8 and 70.5% for the shoulder joint, and 64.0 and 66.7% for the elbow joint, respectively, with an odds ratio (95% CI) of 2.58 (1.03 to 6.46) in the hip joint. The patient global assessment score, SF-36 physical component summary score, EQ-5D QOL score, and acetaminophen consumption for patients with hip joint OA were also better in the DF-HA group than in the placebo group, whereas the improvement with DF-HA was equivocal for all endpoints for all other joints (Table 3, and Supplementary Table 7 and Supplementary Table 8, Additional file 7 and Additional file 8).

**Table 2:** Secondary outcome: joint-specific questionnaires over 12 weeks

Joint	Joint-specific questionnaires	LSM change from baseline (95% CI)		
		DF-HA	Placebo	Difference
Hip	*N*	46	44	
(WOMAC^a^)	Pain subscore (mm)	−26.5 (−35.1 to −18.0)	−17.3 (−26.8 to −7.8)	−9.2 (−17.1 to − 1.4)
	Stiffness subscore (mm)	−27.7 (−36.3 to −19.1)	−20.0 (− 29.6 to − 10.4)	−7.8 (− 15.5 to 0.0)
	Physical function subscore (mm)	−27.3 (−35.7 to −18.8)	−17.4 (−26.8 to −8.0)	−9.9 (−17.5 to −2.3)
	Total score (mm)	−27.1 (−35.3 to −18.8)	−17.4 (−26.5 to −8.2)	−9.7 (−17.2 to −2.2)
Ankle	*N*	30	30	
(SAFE-Q^b^)	Pain and pain-related	21.3 (15.7 to 26.8)	21.3 (15.3 to 27.3)	−0.1 (−6.6 to 6.5)
	Physical functioning and daily living	16.7 (11.0 to 22.5)	13.1 (6.8 to 19.3)	3.7 (−3.1 to 10.5)
	Social functioning	9.9 (2.5 to 17.4)	7.7 (−0.4 to 15.8)	2.2 (−6.8 to 11.3)
	Shoe-related	10.6 (3.6 to 17.6)	4.4 (−2.8 to 11.6)	6.2 (−2.3 to 14.7)
	General health and well-being	14.9 (6.5 to 23.3)	14.7 (5.4 to 24.0)	0.2 (−9.9 to 10.3)
	Sports activity^c^	4.1 (−19.5 to 27.6)	−10.7 (−67.7 to 46.4)	14.7 (−38.5 to 67.9)
Shoulder	*N*	45	45	
(Shoulder36^b^)	Pain	0.38 (0.23 to 0.53)	0.41 (0.25 to 0.57)	−0.03 (−0.23 to 0.16)
	Range of motion	0.44 (0.28 to 0.60)	0.44 (0.27 to 0.60)	0.00 (−0.20 to 0.20)
	Muscle strength	0.60 (0.41 to 0.80)	0.53 (0.33 to 0.72)	0.08 (−0.17 to 0.32)
	General health	0.28 (0.13 to 0.42)	0.36 (0.21 to 0.51)	−0.09 (− 0.27 to 0.10)
	Activities of daily living	0.45 (0.29 to 0.61)	0.39 (0.23 to 0.56)	0.05 (− 0.15 to 0.26)
	Ability to play sports	0.42 (0.20 to 0.63)	0.49 (0.27 to 0.71)	−0.07 (− 0.35 to 0.21)
Elbow	*N*	25	25	
(PREE-J^a^)	Pain score	−12.7 (−17.5 to −7.9)	− 15.4 (−20.2 to − 10.6)	2.7 (−2.4 to 7.8)
	Function score	−5.2 (− 10.0 to − 0.3)	−9.7 (−14.4 to − 5.0)	4.5 (−0.2 to 9.3)
	Specific activities	− 9.7 (− 20.5 to 1.1)	−21.2 (−31.7 to −10.8)	11.5 (1.1 to 22.0)
	Usual activities	−5.0 (−9.2 to −0.9)	−7.3 (−11.3 to −3.3)	2.3 (−1.8 to 6.4)
	Total score	−17.0 (−26.5 to −7.4)	−25.1 (−34.4 to −15.9)	8.2 (−1.4 to 17.8)

*LSM* least-squares means, *CI* confidence interval, *DF-HA* diclofenac etalhyaluronate, *WOMAC* Western Ontario and McMaster Universities Osteoarthritis 3.1 index, *SAFE-Q* Self-Administered Foot Evaluation Questionnaire, *PREE-J* Patient-Rated Elbow Evaluation, Japanese Version

^a^Higher scores indicate more pain or functional disability, and lower scores indicate less pain or functional disability

^b^Lower scores indicate more pain or functional disability, and higher scores indicate less pain or functional disability

^c^The questionnaires were required to be answered only by patients who engaged in sports activity (DF-HA: *n* = 11, Placebo: *n* = 3)

**Table 3:** Other secondary outcomes over 12 weeks

Joint	Joint-specific questionnaires	LSM change from baseline (95% CI)		
		DF − HA	Placebo	Difference
Hip	*N*	46	44	
	Patient global assessment score (mm)^a^	−35.5 (−44.5 to −26.5)	−25.7 (−35.7 to −15.6)	−9.9 (− 17.9 to − 1.8)
	Physician global assessment score (mm)^a^	− 27.6 (−35.3 to −19.8)	−23.8 (−32.3 to −15.2)	−3.8 (−10.6 to 3.0)
	SF-36 summary score			
	MCS	4.5 (1.8 to 7.3)	4.6 (1.6 to 7.7)	−0.1 (−2.6 to 2.4)
	RCS	3.7 (−1.1 to 8.5)	1.0 (−4.3 to 6.2)	2.8 (−1.5 to 7.1)
	PCS	4.9 (0.3 to 9.4)	0.8 (−4.3 to 5.8)	4.1 (0.0 to 8.3)
	EQ-5D			
	QOL score	0.13 (0.07 to 0.19)	0.06 (−0.00 to 0.13)	0.07 (0.02 to 0.12)
	VAS score	17.4 (8.6 to 26.2)	11.0 (1.1 to 20.9)	6.4 (−1.8 to 14.6)
Ankle	*N*	30	30	
	Patient global assessment score (mm)^a^	−21.7 (−29.9 to −13.4)	−21.3 (−30.3 to −12.3)	−0.3 (−10.4 to 9.7)
	Physician global assessment score (mm)^a^	−25.0 (−32.1 to −18.0)	−24.2 (−32.1 to −16.3)	−0.8 (−9.5 to 7.8)
	SF-36 summary score			
	MCS	3.7 (0.8 to 6.7)	2.8 (−0.3 to 5.9)	0.9 (−2.6 to 4.4)
	RCS	−5.4 (−10.8 to 0.0)	−2.9 (−8.8 to 3.0)	−2.5 (−9.0 to 4.0)
	PCS	−0.3 (−4.9 to 4.3)	−2.6 (−7.4 to 2.1)	2.4 (−3.2 to 7.9)
	EQ-5D			
	QOL score	0.06 (0.01 to 0.12)	0.06 (−0.00 to 0.12)	0.00 (−0.06 to 0.07)
	VAS score	1.6 (−5.4 to 8.6)	1.2 (−6.4 to 8.8)	0.4 (−8.0 to 8.9)
Shoulder	N	45	45	
	Patient global assessment score (mm)^a^	−20.2 (−25.3 to −15.1)	−20.1 (− 25.3 to −14.8)	−0.1 (− 6.7 to 6.4)
	Physician global assessment score (mm)^a^	−20.5 (− 25.6 to − 15.4)	−19.9 (− 25.2 to − 14.7)	−0.5 (−7.1 to 6.0)
	SF-36 summary score			
	MCS	3.3 (0.9 to 5.6)	2.8 (0.4 to 5.2)	0.5 (−2.5 to 3.5)
	RCS	−1.4 (−4.9 to 2.2)	−0.2 (−3.9 to 3.5)	−1.2 (−5.7 to 3.3)
	PCS	0.3 (−2.6 to 3.2)	2.3 (−0.7 to 5.3)	−2.0 (−5.7 to 1.7)
	EQ-5D			
	QOL score	0.06 (0.02 to 0.09)	0.08 (0.05 to 0.12)	−0.03 (−0.07 to 0.02)
	VAS score	5.0 (−0.3 to 10.4)	8.1 (2.7 to 13.6)	−3.1 (−9.8 to 3.7)
Elbow	*N*	25	25	
	Patient global assessment score (mm)^a^	−26.2 (−37.5 to −14.9)	−29.9 (−41.1 to −18.8)	3.7 (−7.6 to 15.0)
	Physician global assessment score (mm)^a^	−20.1 (−30.1 to −10.1)	−25.8 (−35.8 to −15.9)	5.7 (−4.1 to 15.6)
	SF-36 summary score			
	MCS	1.4 (−2.9 to 5.8)	3.7 (−0.6 to 8.0)	−2.3 (−6.6 to 2.0)
	RCS	−5.6 (−10.5 to −0.8)	−1.9 (−6.7 to 3.0)	−3.8 (−8.6 to 1.1)
	PCS	4.6 (−0.4 to 9.6)	1.0 (−3.8 to 5.9)	3.6 (−1.4 to 8.6)
	EQ-5D			
	QOL score	0.02 (−0.05 to 0.09)	0.06 (−0.01 to 0.13)	−0.04 (− 0.11 to 0.04)
	VAS score	1.8 (−6.5 to 10.2)	3.9 (−4.4 to 12.3)	−2.1 (−10.6 to 6.4)

*LSM* least-squares means, *CI* confidence interval, *DF-HA* diclofenac etalhyaluronate, *SF-36* Medical Outcomes Study 36-Item Short Form Health Survey, *MCS* mental component summary, *RCS* role/social component summary, *PCS* physical component summary, *EQ-5D* EuroQol 5 Dimensions, *QOL* quality of life, *VAS* visual analog scale

^a^Average of 7 days prior to Week 0 by the 0–100 mm visual analog scale for pain intensity: 0 mm indicates no pain, and 100 mm indicates the worst pain

### Safety

The incidence of TEAEs for all four joints combined was 49.3% (72/146) in the DF-HA group and 36.1% (52/144) in the placebo group (Table 4). No severe TEAEs were reported in either treatment group. Serious TEAEs were ischemic colitis in 1 subject with hip OA, cerebellar hemorrhage in 1 subject with shoulder OA, and radius fracture in 1 subject with elbow OA in the DF-HA group, and subdural hematoma in 1 subject with shoulder OA in the placebo group. All of these serious TEAEs were considered moderate in severity and improved or resolved during the study; none were related to the study drug, and no TEAEs led to treatment discontinuation in either treatment group. Common TEAEs reported in ≥2% of subjects were nasopharyngitis (coded from common cold), injection site joint pain, nausea, palpitations, and arthralgia. TEAEs at the injection site were reported in 10 subjects (6.8%) in the DF-HA group and 7 subjects (4.9%) in the placebo group. There were few TEAEs related to gastrointestinal disorders, cardiovascular disorders, or renal dysfunction, which are characteristic to NSAIDs, and there were no differences between the treatment groups in TEAEs related to hypersensitivity or anaphylactic reaction, which were reported in clinical studies of DF-HA in patients with knee OA.

**Table 4:** Overview of treatment-emergent adverse events

	Hip		Ankle		Shoulder		Elbow		Total	
	DF-HA *N* = 46	Placebo *N* = 44	DF-HA *N* = 30	Placebo *N* = 30	DF-HA *N* = 45	Placebo *N* = 45	DF-HA *N* = 25	Placebo *N* = 25	DF-HA *N* = 146	Placebo *N* = 144
All events	24 (52.2)	15 (34.1)	15 (50.0)	10 (33.3)	23 (51.1)	18 (40.0)	10 (40.0)	9 (36.0)	72 (49.3)	52 (36.1)
Severity										
Mild	23 (50.0)	13 (29.5)	15 (50.0)	9 (30.0)	20 (44.4)	17 (37.8)	8 (32.0)	8 (32.0)	66 (45.2)	47 (32.6)
Moderate	1 (2.2)	2 (4.5)	0	1 (3.3)	3 (6.7)	1 (2.2)	2 (8.0)	1 (4.0)	6 (4.1)	5 (3.5)
Severe	0	0	0	0	0	0	0	0	0	0
Serious events	1 (2.2)	0	0	0	1 (2.2)	1 (2.2)	1 (4.0)	0	3 (2.1)	1 (0.7)
Events leading to study drug withdrawal	0	0	0	0	0	0	0	0	0	0
Common events (≥ 2%)^a^										
Nasopharyngitis	8 (17.4)	4 (9.1)	6 (20.0)	2 (6.7)	5 (11.1)	5 (11.1)	2 (8.0)	2 (8.0)	21 (14.4)	13 (9.0)
Injection site joint pain	3 (6.5)	1 (2.3)	2 (6.7)	2 (6.7)	1 (2.2)	0	0	0	6 (4.1)	3 (2.1)
Nausea	4 (8.7)	0	1 (3.3)	0	0	0	0	0	5 (3.4)	0
Palpitations	2 (4.3)	0	0	0	1 (2.2)	0	0	0	3 (2.1)	0
Arthralgia	0	0	1 (3.3)	2 (6.7)	0	2 (4.4)	0	0	1 (0.7)	4 (2.8)
Special-interest events										
Events at the injection site^b^	3 (6.5)	2 (4.5)	4 (13.3)	3 (10.0)	3 (6.7)	0	0	2 (8.0)	10 (6.8)	7 (4.9)
Gastrointestinal disorders^c^	0	0	0	0	1 (2.2)	0	0	0	1 (0.7)	0
Cardiovascular disorders^d^	2 (4.3)	0	0	0	1 (2.2)	0	0	0	3 (2.1)	0
Renal dysfunction^e^	0	0	0	0	1 (2.2)	0	0	0	1 (0.7)	0
Hypersensitivity^f^	1 (2.2)	0	1 (3.3)	1 (3.3)	3 (6.7)	3 (6.7)	0	1 (4.0)	5 (3.4)	5 (3.5)
Anaphylactic reaction^g^	3 (6.5)	0	1 (3.3)	0	1 (2.2)	2 (4.4)	0	2 (8.0)	5 (3.4)	4 (2.8)

Data are presented as n (%)

*DF-HA* diclofenac etalhyaluronate

Classifications of adverse events are according to the Medical Dictionary for Regulatory Activities (MedDRA), version 22.0

^a^Treatment-emergent adverse events (TEAEs) that occurred at a frequency of ≥2% in the DF-HA and/or placebo groups in total joints

^b^Injection-site events included joint pain, bruising, eczema, hypoesthesia, injury, joint swelling, musculoskeletal pain, joint space narrowing, and cubital tunnel syndrome

^c^Standardized MedDRA query (broad scope) term. TEAEs indicate gastrointestinal perforation, ulceration, hemorrhage, or obstruction

^d^Standardized MedDRA query (broad scope) term. TEAEs indicate acute cardiac failure, ischemic heart disease, or cardiac arrhythmias

^e^Standardized MedDRA query (broad scope) term. TEAEs indicate acute renal failure or chronic kidney disease

^f^Standardized MedDRA query (broad scope) term. TEAEs indicate hypersensitivity

^g^Standardized MedDRA query (broad scope) term. TEAEs indicate anaphylactic reaction

No radiographic changes were considered clinically significant. However, radiographic change in one subject with shoulder OA in the DF-HA group was considered a TEAE of mild joint space narrowing not requiring treatment. In addition, radiographic changes considered joint space narrowing were observed in 5 subjects with hip OA (10.9%) only in the DF-HA group; however, because the narrowing was unchanged from before study entry and without clinical symptoms in all subjects, none of these changes were considered as a TEAE (Supplementary Table 9, Additional file 9). In the manual joint examination, there were no differences between the groups in the frequency of subjects whose joint symptoms worsened from baseline (Supplementary Table 10, Additional file 10), and there were no clinically significant findings. No clinically significant changes were observed in laboratory test results or vital signs.

## Discussion

The efficacy and safety of DF-HA were evaluated in patients with OA of the hip, ankle, shoulder, or elbow who received a total of three intra-articular injections of 30 mg of DF-HA every 4 weeks. In OA of the ankle, shoulder, and elbow, no improvement was observed for any endpoints in the DF-HA group compared with the placebo group. However, in hip OA, statistically significant improvement was observed not only in mean change from baseline in NRS for pain over 12 weeks after the first injection, but also for WOMAC and other endpoints in the DF-HA group compared with the placebo group. By time point, improvement in pain symptoms was observed from Weeks 1–12. No clinically significant adverse events were reported in any joint with OA.

In the current study, which was conducted to determine whether DF-HA is as effective for non-knee OA as for knee OA, the efficacy differed among the affected joints. DF-HA, which has been shown to be effective for knee OA [12], tended to produce better outcomes in weight-bearing joints than in non-weight-bearing joints. The differences in outcomes between the joints may be partly explained by differences in load on each joint in daily life, which may have affected the pain assessment. It is recommended that patients with a certain intensity of pain be included in a study to assess pain appropriately [26, 27]. However, the load on a joint differs between joints depending on the presence or absence of burden and the frequency of joint use. While the main load on weight-bearing joints is burden load arising from activities of daily living, such as standing and walking, the main load on non-weight-bearing joints is workload arising from using an affected joint. Therefore, given the differences in the criteria for pain intensity and worst pain between weight-bearing and non-weight-bearing joints, the efficacy of DF-HA for the non-weight-bearing joints may not have been evaluated appropriately. Equivocal outcomes of ankle OA compared with those of hip or knee OA may be explained by the susceptibility of the ankle joint to direct impact of floor reaction force, the existence of adjacent joints that assist in maintaining ankle functions, such as foot range of motion and changing behavior, and the variability of impact on the joint owing to changes in behavior that result in fluctuations in daily pain in patients with ankle OA, all of which may have affected the study results.

Another possible cause of the different responses between joints is the difference in the difficulty of injection. Compared with the knee joint, which is relatively easy to inject, the joints evaluated in the present study are difficult to inject accurately without guidance because of their structural complexity [28–31]. Blind injection was used in some joints, except for the hip joint, for which ultrasound or fluoroscopy-guided injection was mandatory. This difference in the accuracy of injection may have affected the results, and it may be important to give a guided intra-articular injection in a reliable manner. In addition, differences in joint size and biomechanics, the placebo effect owing to the procedural invasiveness, and the physiological effect may have affected the differences in outcome among the joints.

Regarding safety, no clinically significant TEAEs were reported in this study. Common TEAEs (≥ 2%) were similar to those in two studies in patients with knee OA [11, 12], with no joint-specific events observed. Radiography revealed a TEAE of mild joint space narrowing in the shoulder joint. Joint space narrowing was also observed in the hip joint, although this was not considered a TEAE by the investigator. NSAIDs have been reported to have an adverse effect on cartilage metabolism to varying degrees [32, 33], and further studies may thus be needed to determine the effect of DF-HA on cartilage and other joint structures because the observation period in the current study was as short as 12 weeks, and the sample size was limited in a study evaluating the safety of long-term treatment with DF-HA [34]. In addition, a certain number of local TEAEs were reported in the treated joints, but there were no differences in the incidence between the treatment groups or between the joints. As mentioned, injecting joints other than the knee is often more challenging. In the current study, there were no events of concern attributable to an injection procedure; however, the sample size was small for each joint studied. Caution may be needed in the injection procedure.

DF-HA was developed as a novel intra-articular injection agent of HA linked with DF. Existing intra-articular injection agents are primarily recommended for use in the knee [1, 3]. From the results of this study, although the mean change from baseline in WOMAC and patient global assessment score over 12 weeks of DF-HA and placebo group were improved over the minimal clinically important improvement [35], DF-HA was statistically significantly better than placebo. Therefore, DF-HA is expected to be effective for hip OA and provides a new therapeutic option. While HA has been reported effective for hip OA [36–39], DF-HA may be clinically advantageous in that it relieves pain immediately after injection.

This study had some limitations. 1) The sample size was limited for each joint studied, and further studies should thus be conducted to increase the evidence for the efficacy of DF-HA for hip and ankle OA. 2) Four joints were evaluated in a single study, and the study design may have been inappropriate for some joints. In particular, the efficacy of DF-HA for shoulder and elbow OA should be re-evaluated in a well-designed study. 3) The observation period was as short as 12 weeks. Because some patients may receive DF-HA long-term, the long-term safety of extended treatment should be evaluated. 4) OA involves multiple pathological mechanisms, including articular cartilage, subchondral bone, and synovium change, and long-term safety studies using further imaging techniques, such as magnetic resonance imaging, might be necessary.

## Conclusions

Intra-articular DF-HA produced a rapid response and was safe in patients with hip OA, with analgesia maintained for 12 weeks when administered every 4 weeks.

## Supplementary Information

**Additional file 1: Supplementary Table 1.** Injection technique.

**Additional file 2: Supplementary Table 2.** Adverse events (AEs): definitions for evaluating and reporting.

**Additional file 3: Supplementary Table 3.** X-ray evaluation method.

**Additional file 4: Supplementary Table 4.** Patients’ demographic and baseline characteristics (each treatment group).

**Additional file 5: Supplementary Table 5.** Change from baseline in NRS for pain at each time point.

**Additional file 6: Supplementary Table 6.** Responder rate at each time point.

**Additional file 7: Supplementary Table 7.** Change from baseline in acetaminophen consumption at each time point.

**Additional file 8: Supplementary Table 8.** Change from baseline in range of motion at Week 12.

**Additional file 9: Supplementary Table 9.** Structural changes visible by X-ray imaging at the last assessment.

**Additional file 10: Supplementary Table 10.** Symptoms of worsening during target joint examinations.

## Data Availability

The datasets generated and analyzed during the current study are available from the corresponding author on reasonable request.

## Abbreviations

CI: Confidence interval
DF: Diclofenac sodium
DF-HA: Diclofenac etalhyaluronate
EQ-5D: EuroQol 5 Dimensions
FAS: Full analysis set
GCP: Good Clinical Practice
GEE: Generalized estimating eq.
HA: Hyaluronic acid
IRB: Institutional review board
KL: Kellgren–Lawrence
MedDRA: Medical Dictionary for Regulatory Activities
MMRM: Mixed model for repeated measures
NRS: Numerical rating scale
NSAIDs: Non-steroidal anti-inflammatory drugs
OA: Osteoarthritis
PREE-J: Patient-Rated Elbow Evaluation (Japanese Version)
QOL: Quality of life
SAFE-Q: Self-Administered Foot Evaluation Questionnaire
SF-36: Medical Outcomes Study 36-Item Short Form Health Survey
TEAE: Treatment-emergent adverse event
VAS: Visual analog scale
WOMAC: Western Ontario and McMaster Universities Osteoarthritis 3.1 index
