# Supplementary material for: Diclofenac–hyaluronate conjugate (diclofenac etalhyaluronate) intra-articular injection for hip, ankle, shoulder, and elbow osteoarthritis: a randomized controlled trial
Source: BMC Musculoskelet Disord. 2022 Apr 20;23:371. doi: 10.1186/s12891-022-05328-3 (PMC9022275; doi:10.1186/s12891-022-05328-3)
Supplement: Supplementary file 1 — Additional file 1: Supplementary Table 1. Injection technique. [file 12891_2022_5328_MOESM1_ESM.docx]

Additional file 1: Supplementary Table 1 Injection technique.

| Joint | Injection Technique |
| --- | --- |
| Hip | In principle, administration is performed using a 21-G, 22-G or 23-G needle, either a 60- or 70-mm-long Cattelan needle, or an 89-mm-long lumbar puncture needle.  <Echographically guided hip intra-articular administration>   - The subject is placed in the supine position. - The probe is rotated through approximately 45º in relation to the femoral axis, and the hip joint is viewed in the longitudinal direction of the femoral neck. - Approaching from the anterior or anterolateral direction, the syringe needle is inserted, aiming at the center of the femoral neck, and the entire dose of 3 mL of study drug is injected.   <X-radiographically guided hip intra-articular administration>   - The subject is placed in the supine position. - Approaching from the anterior or anterolateral direction, the syringe needle is inserted, aiming at the center of the femoral neck, and then moved forward with X-radiographic guidance. - When it has been confirmed X-radiographically that the needle tip has reached the joint space, the entire dose of 3 mL of study drug is injected. |
| Ankle | In principle, administration is performed using a 22-G or 23-G needle, either a 32- or 38-mm-long syringe needle, or a 60-mm-long Cattelan needle.  <Echographically guided ankle intra-articular administration>   - With the subject in a semi-supine position, his/her knee is gently bent, an object such as a pillow or towel is placed behind the ankle joint, and the heel is thus kept unsupported. This position is to correct anterior subluxation of the calcaneus and talus. - The probe is moved forward in the direction of the ankle joint longitudinal axis, and the anterior tibial tendon and talocrural joint are viewed. - The syringe needle is inserted immediately to the medial side of the anterior tibial tendon, and is moved forward in such a way as to be parallel with the superior surface of the talus. - The needle is pushed forward to a deep position, and when it has entered inside the joint capsule the entire dose of 3 mL of study drug is injected.   <X-radiographically guided ankle intra-articular administration>   - With the subject in a semi-supine position, his/her knee is gently bent, an object such as a pillow or towel is placed behind the ankle joint, and the heel is thus kept unsupported. This position is to correct anterior subluxation of the calcaneus and talus. - The anterior surface of the ankle joint space is palpated, and the appropriate position for insertion into the ankle joint is decided upon. In general, a position immediately to the medial side of the anterior tibial tendon is appropriate. - The syringe needle is moved forward from the insertion site, and is then moved forward in such a way as to be parallel with the superior surface of the talus, with X-radiographic guidance. - When it is X-radiographically confirmed that the needle tip has entered the joint capsule, the entire dose of 3 mL of study drug is injected.   <Blind ankle intra-articular administration>   - With the subject in a semi-supine position, his/her knee is gently bent, an object such as a pillow or towel is placed behind the ankle joint, and the heel is thus kept unsupported. This position is to correct anterior subluxation of the calcaneus and talus. - The anterior surface of the ankle joint space is palpated, and the appropriate position for insertion into the ankle joint is decided upon. In general, a position immediately to the medial side of the anterior tibial tendon is appropriate. - The syringe needle is moved forward from the insertion site, and then in such a way as to be parallel with the superior surface of the talus. - The needle is moved forward to a deep position, and, when it is confirmed that it has entered the joint capsule, the entire dose of 3 mL of study drug is injected. |
| Shoulder | In principle, administration is performed using a 21-G or 22-G needle, either a 38-mm-long syringe needle or a 60-mm-long Cattelan needle.  <Echographically guided shoulder intra-articular administration>  The procedure by a posterior approach, which is a representative administration method, is as follows:   - While in the seated position, the subject lets his/her upper arm hang down, and rotates his/her shoulder a little. - Approaching from the rear, the probe is used to view the glenohumeral joint. - The needle is inserted by the cross-over method, aiming at the lateral side of the joint space. - When the needle tip enters the joint, the drug solution is injected.   <X-radiographically guided shoulder intra-articular administration>  The procedure by an anterior approach, which is a representative administration method, is as follows:   - With the subject in the seated or supine position, the coracoid process is palpated. - With the lower lateral side of the coracoid process as the puncture site, the needle is moved toward the shoulder joint, under X-radiographic observation. - The needle is moved forward, and, when entry of the needle tip into the joint space is confirmed X-radiographically, the entire dose of 3 mL of study drug is injected.   <Blind shoulder intra-articular administration>  The procedure by anterior and posterior approaches, which are representative administration methods, are as follows:  **Posterior approach**   - With the subject in the seated or prone position, he/she bends his/her elbow, lays his/her arm in front of his/her waist, and rotates his/her shoulder joint externally, so as to open a route for access to the shoulder joint posterior surface. - With a locus somewhat medial to and below the acromial angle as the puncture site, the coracoid process is touched with the index finger, and insertion is performed aiming at that point. - The needle is slowly pushed forward, and when it is felt to first pass through the joint capsule, and then stop at the shoulder joint surface, the entire dose of 3 mL of study drug is injected. - If resistance is felt while the syringe is being pushed in, it is possible that the needle tip has come into contact with joint cartilage, so the needle is withdrawn slightly, and the study drug is then injected.   **Anterior approach**   - With the subject in the seated or supine position, the coracoid process is palpated. - With the lower lateral side of the coracoid process as the puncture site, the needle is moved forward toward the shoulder joint. - The needle is slowly pushed forward, and when it is felt to first pass through the joint capsule, and then stop at the shoulder joint surface, the entire dose of 3 mL of study drug is injected. |
| Elbow | In principle, administration is performed using a 22-G or 23-G needle, either a 32-mm-long syringe needle or a 60-mm-long Cattelan needle.  <Echographically guided elbow intra-articular administration>  The procedure by a posterior approach, which is a representative administration method, is as follows:   - While in the seated position, the subject bends his/her elbow through approximately 90º. - Approaching from the rear, the probe is used to view the elbow joint. - By the parallel method, the needle is passed through the triceps brachii. - The needle is inserted into the joint space between the olecranon fossa and tip, and the entire dose of 3 mL of study drug is injected.   <X-radiographically guided elbow intra-articular administration>  The procedure by a lateral approach, which is a representative administration method, is as follows:   - While in the seated position, the subject bends his/her elbow through approximately 45º, followed by pronation of the forearm. - The radius is palpated at the posterior lateral surface of the elbow joint, and the position of the humeroradial joint space is confirmed. A visual marker is placed in the central part of the joint space. - The needle is inserted between the radial head and the humeral capitellum, and moved forward with X-radiographic guidance. - When it is confirmed X-radiographically that the needle tip has entered the joint space, the entire dose of 3 mL of study drug is injected. - Care must be taken not to damage the elbow joint cartilage during insertion.   <Blind elbow intra-articular administration>  The procedure by the lateral approach, which is a representative administration method, is as follows:   - While in the seated position, the subject bends his/her elbow through approximately 45º, followed by pronation of the forearm. - The radius is palpated at the posterior lateral surface of the elbow joint, and the position of the humeroradial joint space is confirmed. A visual marker is placed in the central part of the joint space. - The needle is inserted between the radial head and the humeral capitellum, and the entire dose of 3 mL of study drug is injected. - Care must be taken not to damage the elbow joint cartilage during insertion. |
