# Supplementary material for: Diclofenac–hyaluronate conjugate (diclofenac etalhyaluronate) intra-articular injection for hip, ankle, shoulder, and elbow osteoarthritis: a randomized controlled trial
Source: BMC Musculoskelet Disord. 2022 Apr 20;23:371. doi: 10.1186/s12891-022-05328-3 (PMC9022275; doi:10.1186/s12891-022-05328-3)
Supplement: Supplementary file 2 — Additional file 2: Supplementary Table 2. Adverse events (AEs): definitions for evaluating and reporting. [file 12891_2022_5328_MOESM2_ESM.docx]

Additional file 2: Supplementary Table 2 Adverse events (AEs): definitions for evaluating and reporting.

| Item | Definition |
| --- | --- |
| AE | An AE is any unfavorable or unintended sign (including laboratory test abnormalities), symptom, or disease occurring after obtaining consent, regardless of the causal relationship with the study drug. |
| Serious AEs (SAE) | An SAE is an AE characterized by any of the following:   1. Results in death 2. Is life-threatening   “Life-threatening” means that the patient was at risk of dying at the time that the event occurred, not that the event could hypothetically have caused death if it had been more severe.   1. Requires in-patient hospitalization or prolongation of existing hospitalization for treatment 2. Results in persistent or significant disability or incapacity 3. Causes a congenital abnormality or birth defect 4. Any other medically significant event or reaction   Medical and scientific judgment should be exercised when deciding whether other situations should be considered serious, such as important medical events that might not be immediately life-threatening or result in death or hospitalization but might jeopardize the patient or might require intervention to prevent one of the other outcomes listed in the definition above. Examples of such events are intensive treatment in an emergency room or at home for allergic bronchospasm, blood dyscrasias, or convulsions that do not result in hospitalization, or development of drug dependency or drug abuse. |
| Assessment of severity | AE severity is graded according to the following grades:  Mild: does not or only minimally interferes with activities of daily living. No intervention or only simple intervention indicated.  Moderate: interferes with activities of daily living; intervention indicated.  Severe: can perform almost no activities of daily living, or systemic intervention indicated. |
| Assessment of causality | A treatment-related AE is an AE for which causality with the study drug is at least a reasonable possibility; i.e., a causal relationship cannot be ruled out.  Causal relationship with the study drug is assessed using the following guidelines:  Related: There is at least a reasonable possibility that the event was due to the study drug, and a causal relationship cannot be ruled out.  Not related: The event does not have a temporal relationship with study drug administration and can be explained by another factor, such as underlying disease, complications, concomitant medications, diathesis, or concomitant therapies. |
