# Supplementary material for: Diclofenac–hyaluronate conjugate (diclofenac etalhyaluronate) intra-articular injection for hip, ankle, shoulder, and elbow osteoarthritis: a randomized controlled trial
Source: BMC Musculoskelet Disord. 2022 Apr 20;23:371. doi: 10.1186/s12891-022-05328-3 (PMC9022275; doi:10.1186/s12891-022-05328-3)
Supplement: Supplementary file 3 — Additional file 3: Supplementary Table 3. X-ray evaluation method. [file 12891_2022_5328_MOESM3_ESM.docx]

**Additional file 3:** **Supplementary Table 3** X-ray evaluation method.

| Item | Method |
| --- | --- |
| Osteophyte | Evaluation on a 5-item scale (Normal, Possible, Definite, Moderate, Large) according to X-ray imaging. |
| Joint space narrowing | Evaluation on a 5-item scale (Normal, Doubtful, Questionable, Definite, Marked) according to X-ray imaging. |
| Osteosclerosis | Evaluation on a 3-item scale (Normal, Some, Severe) according to X-ray imaging. |
| Epiphyseal deformity | Evaluation on a 3-item scale (Normal, Possible, Definite) according to X-ray imaging. |
