# Supplementary material for: Diclofenac–hyaluronate conjugate (diclofenac etalhyaluronate) intra-articular injection for hip, ankle, shoulder, and elbow osteoarthritis: a randomized controlled trial
Source: BMC Musculoskelet Disord. 2022 Apr 20;23:371. doi: 10.1186/s12891-022-05328-3 (PMC9022275; doi:10.1186/s12891-022-05328-3)
Supplement: Supplementary file 4 — Additional file 4: Supplementary Table 4. Patients’ demographic and baseline characteristics (each treatment group). [file 12891_2022_5328_MOESM4_ESM.docx]

**Additional file 4:** **Supplementary Table 4** Patients’ demographic and baseline characteristics (each treatment group).

| Characteristic | Hip | | Ankle | | Shoulder | | Elbow | | Total | |
| --- | --- | --- | --- | --- | --- | --- | --- | --- | --- | --- |
|  | DF-HA  *N* = 46 | Placebo  *N* = 44 | DF-HA  *N* = 30 | Placebo  *N* = 30 | DF-HA  *N* = 45 | Placebo  *N* = 45 | DF-HA  *N* = 25 | Placebo  *N* = 25 | DF-HA  *N* = 146 | Placebo  *N* = 144 |
| Age, years | 61.3 ± 9.6 | 58.5 ± 7.9 | 66.7 ± 12.0 | 64.0 ± 11.2 | 69.0 ± 12.9 | 71.4 ± 9.7 | 61.5 ± 13.1 | 61.1 ± 13.2 | 64.8 ± 12.2 | 64.1 ± 11.4 |
| Sex |  |  |  |  |  |  |  |  |  |  |
| Male | 5 (10.9) | 5 (11.4) | 7 (23.3) | 6 (20.0) | 23 (51.1) | 24 (53.3) | 18 (72.0) | 19 (76.0) | 53 (36.3) | 54 (37.5) |
| Female | 41 (89.1) | 39 (88.6) | 23 (76.7) | 24 (80.0) | 22 (48.9) | 21 (46.7) | 7 (28.0) | 6 (24.0) | 93 (63.7) | 90 (62.5) |
| BMI, kg/m^2^ | 23.25 ± 3.10 | 23.61 ± 3.66 | 23.98 ± 3.17 | 25.37 ± 3.80 | 24.02 ± 3.69 | 24.04 ± 3.25 | 24.92 ± 3.29 | 23.72 ± 3.61 | 23.92 ± 3.35 | 24.13 ± 3.58 |
| Duration of current joint pain, weeks | 225.3 ± 254.8 | 259.1 ± 258.4 | 175.4 ± 184.0 | 201.1 ± 209.8 | 170.4 ± 206.9 | 152.3 ± 165.2 | 393.8 ± 441.7 | 323.2 ± 363.2 | 227.0 ± 280.3 | 224.8 ± 251.7 |
| Classification of OA |  |  |  |  |  |  |  |  |  |  |
| Primary | 9 (19.6) | 9 (20.5) | 21 (70.0) | 21 (70.0) | 35 (77.8) | 34 (75.6) | 22 (88.0) | 21 (84.0) | 87 (59.6) | 85 (59.0) |
| Secondary | 37 (80.4) | 35 (79.5) | 9 (30.0) | 9 (30.0) | 10 (22.2) | 11 (24.4) | 3 (12.0) | 4 (16.0) | 59 (40.4) | 59 (41.0) |
| Stage of OA^a^, *n* (%) |  |  |  |  |  |  |  |  |  |  |
| Stage A | 14 (30.4) | 13 (29.5) | 9 (30.0) | 10 (33.3) | 27 (60.0) | 26 (57.8) | 11 (44.0) | 11 (44.0) | 61 (41.8) | 60 (41.7) |
| Stage B | 32 (69.6) | 31 (70.5) | 21 (70.0) | 20 (66.7) | 18 (40.0) | 19 (42.2) | 14 (56.0) | 14 (56.0) | 85 (58.2) | 84 (58.3) |
| NRS for pain^b^, *n* (%) | 6.89 ± 1.08 | 6.99 ± 1.05 | 7.18 ± 0.98 | 6.86 ± 1.14 | 6.50 ± 1.03 | 6.77 ± 1.12 | 6.97 ± 0.89 | 6.42 ± 0.90 | 6.84 ± 1.03 | 6.80 ± 1.07 |
| Shoulder36^c^ |  |  |  |  |  |  |  |  |  |  |
| Pain | - | - | - | - | 2.63 ± 0.72 | 2.82 ± 0.84 | - | - | - | - |
| Range of motion | - | - | - | - | 2.50 ± 0.80 | 2.68 ± 0.83 | - | - | - | - |
| Muscle strength | - | - | - | - | 1.96 ± 0.86 | 2.21 ± 1.10 | - | - | - | - |
| General health | - | - | - | - | 3.04 ± 0.70 | 3.08 ± 0.79 | - | - | - | - |
| Activities of daily living | - | - | - | - | 2.70 ± 0.80 | 2.85 ± 0.88 | - | - | - | - |
| Ability to play sports | - | - | - | - | 1.63 ± 0.80 | 1.52 ± 1.19 | - | - | - | - |
| PREE-J^d^ |  |  |  |  |  |  |  |  |  |  |
| Pain score | - | - | - | - | - | - | 35.2 ± 5.0 | 32.5 ± 5.4 | - | - |
| Function score | - | - | - | - | - | - | 21.1 ± 11.0 | 21.5 ± 9.0 | - | - |
| Specific activities | - | - | - | - | - | - | 43.7 ± 24.6 | 45.6 ± 20.8 | - | - |
| Usual activities | - | - | - | - | - | - | 19.7 ± 9.7 | 18.9 ± 7.2 | - | - |
| Total score | - | - | - | - | - | - | 56.2 ± 14.0 | 54.3 ± 11.8 | - | - |
| WOMAC^d^ |  |  |  |  |  |  |  |  |  |  |
| Pain subscore (mm) | 53.3 ± 18.3 | 46.8 ± 18.8 | - | - | - | - | - | - | - | - |
| Stiffness subscore (mm) | 49.3 ± 25.8 | 47.0 ± 27.7 | - | - | - | - | - | - | - | - |
| Physical function subscore (mm) | 51.5 ± 22.8 | 49.5 ± 20.6 | - | - | - | - | - | - | - | - |
| Total score (mm) | 51.7 ± 21.1 | 48.8 ± 18.9 | - | - | - | - | - | - | - | - |
| SAFE-Q^c^ |  |  |  |  |  |  |  |  |  |  |
| Pain and pain-related | - | - | 40.6 ± 11.1 | 41.1 ± 15.3 | - | - | - | - | - | - |
| Physical functioning and daily living | - | - | 50.5 ± 19.2 | 52.9 ± 18.2 | - | - | - | - | - | - |
| Social functioning | - | - | 59.9 ± 28.3 | 62.8 ± 21.6 | - | - | - | - | - | - |
| Shoe-related | - | - | 63.3 ± 24.0 | 53.3 ± 24.1 | - | - | - | - | - | - |
| General health and well-being | - | - | 58.0 ± 28.9 | 63.7 ± 21.3 | - | - | - | - | - | - |
| Sports activity^e^ | - | - | 35.2 ± 24.0 | 15.1 ± 13.7 | - | - | - | - | - | - |
| Range of motion (deg) |  |  |  |  |  |  |  |  |  |  |
| Forward flexion | - | - | - | - | 107.8 ± 31.0 | 126.2 ± 31.3 | - | - | - | - |
| Abduction | 24.6 ± 10.3 | 25.6 ± 10.7 | - | - | 93.2 ± 32.8 | 99.7 ± 33.4 | - | - | - | - |
| External rotation | - | - | - | - | 29.0 ± 19.3 | 31.2 ± 21.6 | - | - | - | - |
| Internal rotation | 17.4 ± 12.9 | 19.1 ± 12.6 | - | - | 63.2 ± 19.0 | 62.9 ± 23.9 | - | - | - | - |
| Extension | 4.0 ± 9.5 | 7.0 ± 8.0 | 9.0 ± 9.5 | 10.7 ± 7.6 | - | - | −16.1 ± 18.7 | −11.6 ± 11.7 | - | - |
| Flexion | 97.1 ± 13.7 | 100.6 ± 15.6 | 39.2 ± 10.8 | 40.3 ± 13.2 | - | - | 119.9 ± 10.5 | 120.2 ± 14.3 | - | - |
| Pronation | - | - | - | - | - | - | 80.9 ± 10.7 | 79.0 ± 13.1 | - | - |
| Supination | - | - | - | - | - | - | 83.0 ± 11.0 | 80.8 ± 20.7 | - | - |
| Patient global assessment score (mm)^f^ | 64.8 ± 20.4 | 65.5 ± 16.1 | 72.2 ± 11.2 | 67.1 ± 15.5 | 65.2 ± 13.5 | 62.6 ± 17.4 | 67.5 ± 12.6 | 62.0 ± 16.7 | 66.9 ± 15.6 | 64.3 ± 16.4 |
| Physician global assessment score (mm)^f^ | 59.5 ± 17.0 | 62.2 ± 11.6 | 67.6 ± 12.6 | 62.0 ± 14.6 | 63.1 ± 13.6 | 57.9 ± 15.5 | 61.5 ± 12.5 | 62.0 ± 16.4 | 62.6 ± 14.6 | 60.8 ± 14.4 |
| SF-36 summary score |  |  |  |  |  |  |  |  |  |  |
| MCS | 51.3 ± 8.4 | 53.0 ± 8.6 | 52.1 ± 9.0 | 56.0 ± 7.8 | 51.9 ± 9.8 | 51.9 ± 9.1 | 50.7 ± 9.0 | 50.6 ± 10.3 | 51.6 ± 9.0 | 52.9 ± 9.0 |
| RCS | 45.2 ± 14.5 | 48.5 ± 13.9 | 49.9 ± 11.5 | 51.2 ± 12.4 | 51.0 ± 13.5 | 47.8 ± 16.0 | 47.4 ± 16.0 | 49.2 ± 9.2 | 48.3 ± 14.0 | 48.9 ± 13.5 |
| PCS | 30.0 ± 12.0 | 27.5 ± 11.7 | 36.2 ± 12.4 | 29.6 ± 11.0 | 31.7 ± 15.1 | 34.3 ± 13.7 | 36.4 ± 9.3 | 40.8 ± 8.7 | 32.9 ± 12.9 | 32.4 ± 12.6 |
| EQ-5D |  |  |  |  |  |  |  |  |  |  |
| QOL score | 0.65 ± 0.20 | 0.67 ± 0.16 | 0.72 ± 0.13 | 0.72 ± 0.14 | 0.72 ± 0.15 | 0.73 ± 0.13 | 0.69 ± 0.17 | 0.79 ± 0.09 | 0.69 ± 0.17 | 0.72 ± 0.14 |
| VAS score | 59.4 ± 19.8 | 65.9 ± 16.3 | 72.7 ± 14.9 | 72.0 ± 14.5 | 68.3 ± 15.4 | 67.7 ± 18.3 | 69.9 ± 15.2 | 74.1 ± 14.8 | 66.7 ± 17.4 | 69.2 ± 16.5 |
| Mean daily acetaminophen consumption (mg/day) | 308.4 ± 386.4 | 235.7 ± 321.7 | 261.4 ± 377.4 | 237.1 ± 288.0 | 242.9 ± 355.9 | 293.3 ± 399.0 | 216.0 ± 258.2 | 168.0 ± 305.8 | 262.7 ± 354.2 | 242.3 ± 338.3 |
| Data are presented as mean ± standard deviation or *n* (%).  DF-HA: diclofenac etalhyaluronate; BMI: body mass index; OA: osteoarthritis; NRS: numerical rating scale; PREE-J: Patient-Rated Elbow Evaluation, Japanese Version; WOMAC: Western Ontario and McMaster Universities Osteoarthritis 3.1 index; SAFE-Q: Self-Administered Foot Evaluation Questionnaire; SF-36: Medical Outcomes Study 36-Item Short Form Health Survey; MCS: mental component summary; RCS: role/social component summary; PCS: physical component summary; EQ-5D: EuroQol 5 Dimensions; QOL: quality of life; VAS: visual analog scale  ^a^Stage A: Kellgren–Lawrence (KL) grading score for hip OA stage (Early stage); ankle OA stage (Stage 1/ Stage 2); shoulder OA and elbow OA (Grade 2). Stage B: KL grading score for; hip OA stage (Advanced stage); ankle OA stage (Stage 3); shoulder OA and elbow OA (Grade 3).  ^b^Average of 7 days prior to Week 0 by the 0–10 numerical rating scale for pain intensity: 0 indicates no pain, and 10 indicates the worst pain.  ^c^Lower scores indicate more pain or functional disability, and higher scores indicate less pain or functional disability.  ^d^Higher scores indicate more pain or functional disability, and lower scores indicate less pain or functional disability.  ^e^The questionnaires were required to be answered only by patients who engaged in sports activity (DF-HA: *n* = 11, Placebo: *n* = 3).  ^f^Average of 7 days prior to Week 0 by the 0–100 mm visual analog scale for pain intensity: 0 mm indicates no pain, and 100 mm indicates the worst pain. | | | | | | | | | | |
