# Supplementary material for: Diclofenac–hyaluronate conjugate (diclofenac etalhyaluronate) intra-articular injection for hip, ankle, shoulder, and elbow osteoarthritis: a randomized controlled trial
Source: BMC Musculoskelet Disord. 2022 Apr 20;23:371. doi: 10.1186/s12891-022-05328-3 (PMC9022275; doi:10.1186/s12891-022-05328-3)
Supplement: Supplementary file 5 — Additional file 5: Supplementary Table 5. Change from baseline in NRS for pain at each time point. [file 12891_2022_5328_MOESM5_ESM.docx]

**Additional file 5:** **Supplementary Table 5** Change from baseline in NRS for pain at each time point.

| Joint | Week | Change from baseline | | | | vs placebo |  |
| --- | --- | --- | --- | --- | --- | --- | --- |
|  |  | DF-HA | | Placebo | | Difference in LSM (95% CI)  (DF-HA − Placebo) |  |
|  |  | *n* | Mean ± SD | *n* | Mean ± SD |  |  |
| Hip | 1 | 46 | −2.34 ± 1.94 | 44 | −1.56 ± 1.86 | −0.74 (−1.54 to 0.06) |  |
|  | 2 | 46 | −2.55 ± 1.77 | 44 | −1.78 ± 1.85 | −0.73 (−1.50 to 0.04) |  |
|  | 4 | 46 | −2.45 ± 1.61 | 43 | −1.67 ± 1.53 | −0.77 (−1.44 to −0.09) |  |
|  | 6 | 46 | −2.85 ± 1.79 | 43 | −2.06 ± 1.77 | −0.79 (−1.55 to −0.03) |  |
|  | 8 | 46 | −2.71 ± 1.82 | 42 | −2.01 ± 1.79 | −0.70 (−1.47 to 0.07) |  |
|  | 10 | 45 | −3.07 ± 1.77 | 42 | −2.13 ± 1.95 | −0.93 (−1.71 to −0.16) |  |
|  | 12 | 44 | −3.04 ± 1.80 | 42 | −2.01 ± 2.04 | −0.98 (−1.78 to −0.18) |  |
| Ankle | 1 | 30 | −1.79 ± 1.91 | 30 | −1.44 ± 1.55 | −0.40 (−1.34 to 0.54) |  |
|  | 2 | 30 | −1.85 ± 2.01 | 30 | −1.74 ± 2.16 | −0.16 (−1.25 to 0.94) |  |
|  | 4 | 30 | −1.46 ± 1.61 | 30 | −1.85 ± 2.01 | 0.34 (−0.62 to 1.30) |  |
|  | 6 | 29 | −2.55 ± 1.96 | 30 | −2.09 ± 2.16 | −0.44 (−1.53 to 0.65) |  |
|  | 8 | 29 | −2.30 ± 1.89 | 30 | −2.38 ± 2.17 | 0.10 (−0.99 to 1.18) |  |
|  | 10 | 29 | −2.66 ± 2.03 | 29 | −2.74 ± 2.42 | −0.04 (−1.28 to 1.20) |  |
|  | 12 | 29 | −2.53 ± 1.88 | 28 | −2.78 ± 2.39 | 0.14 (−0.99 to 1.28) |  |
| Shoulder | 1 | 45 | −1.03 ± 1.46 | 45 | −1.00 ± 1.29 | −0.13 (−0.72 to 0.46) |  |
|  | 2 | 45 | −1.18 ± 1.71 | 45 | −1.39 ± 1.34 | 0.12 (−0.54 to 0.78) |  |
|  | 4 | 45 | −1.42 ± 1.78 | 44 | −1.45 ± 1.31 | −0.09 (−0.75 to 0.57) |  |
|  | 6 | 45 | −1.75 ± 1.75 | 44 | −2.12 ± 1.64 | 0.24 (−0.46 to 0.95) |  |
|  | 8 | 45 | −1.88 ± 1.90 | 44 | −2.11 ± 1.79 | 0.11 (−0.66 to 0.87) |  |
|  | 10 | 44 | −2.34 ± 1.85 | 44 | −2.71 ± 1.94 | 0.28 (−0.49 to 1.06) |  |
|  | 12 | 44 | −2.45 ± 1.87 | 44 | −3.05 ± 2.02 | 0.53 (−0.27 to 1.32) |  |
| Elbow | 1 | 25 | −1.19 ± 1.34 | 25 | −1.29 ± 1.36 | 0.11 (−0.71 to 0.92) |  |
|  | 2 | 25 | −1.28 ± 1.54 | 25 | −1.88 ± 1.63 | 0.60 (−0.35 to 1.54) |  |
|  | 4 | 25 | −1.25 ± 1.81 | 25 | −2.07 ± 1.85 | 0.82 (−0.26 to 1.90) |  |
|  | 6 | 25 | −1.73 ± 1.67 | 25 | −2.59 ± 1.94 | 0.87 (−0.22 to 1.95) |  |
|  | 8 | 25 | −2.02 ± 1.89 | 25 | −2.60 ± 2.17 | 0.59 (−0.62 to 1.79) |  |
|  | 10 | 25 | −2.21 ± 1.96 | 25 | −3.00 ± 2.18 | 0.79 (−0.43 to 2.01) |  |
|  | 12 | 25 | −2.54 ± 2.04 | 24 | −3.05 ± 2.46 | 0.48 (−0.85 to 1.81) |  |
| DF-HA: diclofenac etalhyaluronate; SD: standard deviation; LSM: least-squares means; CI: confidence interval  Average of pain scores according to the 0–10 numerical rating scale for pain intensity: 0 indicates no pain, and 10 indicates the worst pain. | | | | | | | |
