# Supplementary material for: Diclofenac–hyaluronate conjugate (diclofenac etalhyaluronate) intra-articular injection for hip, ankle, shoulder, and elbow osteoarthritis: a randomized controlled trial
Source: BMC Musculoskelet Disord. 2022 Apr 20;23:371. doi: 10.1186/s12891-022-05328-3 (PMC9022275; doi:10.1186/s12891-022-05328-3)
Supplement: Supplementary file 6 — Additional file 6: Supplementary Table 6. Responder rate at each time point. [file 12891_2022_5328_MOESM6_ESM.docx]

**Additional file 6:** **Supplementary Table 6** Responder rate at each time point.

| Joint | Week | DF-HA | | Placebo | |
| --- | --- | --- | --- | --- | --- |
|  |  | *n* | *n* (%) | *n* | *n* (%) |
| Hip | 1 | 46 | 25 (54.3) | 44 | 17 (38.6) |
|  | 2 | 46 | 23 (50.0) | 44 | 19 (43.2) |
|  | 4 | 46 | 26 (56.5) | 43 | 17 (39.5) |
|  | 6 | 46 | 29 (63.0) | 43 | 19 (44.2) |
|  | 8 | 46 | 27 (58.7) | 42 | 17 (40.5) |
|  | 10 | 45 | 36 (80.0) | 42 | 20 (47.6) |
|  | 12 | 44 | 30 (68.2) | 42 | 19 (45.2) |
| Ankle | 1 | 30 | 12 (40.0) | 30 | 8 (26.7) |
|  | 2 | 30 | 13 (43.3) | 30 | 9 (30.0) |
|  | 4 | 30 | 8 (26.7) | 30 | 12 (40.0) |
|  | 6 | 29 | 13 (44.8) | 30 | 15 (50.0) |
|  | 8 | 29 | 12 (41.4) | 30 | 19 (63.3) |
|  | 10 | 29 | 17 (58.6) | 29 | 17 (58.6) |
|  | 12 | 29 | 15 (51.7) | 28 | 16 (57.1) |
| Shoulder | 1 | 45 | 8 (17.8) | 45 | 8 (17.8) |
|  | 2 | 45 | 12 (26.7) | 45 | 14 (31.1) |
|  | 4 | 45 | 16 (35.6) | 44 | 14 (31.8) |
|  | 6 | 45 | 18 (40.0) | 44 | 23 (52.3) |
|  | 8 | 45 | 20 (44.4) | 44 | 21 (47.7) |
|  | 10 | 44 | 24 (54.5) | 44 | 29 (65.9) |
|  | 12 | 44 | 25 (56.8) | 44 | 31 (70.5) |
| Elbow | 1 | 25 | 5 (20.0) | 25 | 7 (28.0) |
|  | 2 | 25 | 8 (32.0) | 25 | 12 (48.0) |
|  | 4 | 25 | 9 (36.0) | 25 | 13 (52.0) |
|  | 6 | 25 | 10 (40.0) | 25 | 16 (64.0) |
|  | 8 | 25 | 12 (48.0) | 25 | 16 (64.0) |
|  | 10 | 25 | 13 (52.0) | 25 | 17 (68.0) |
|  | 12 | 25 | 16 (64.0) | 24 | 16 (66.7) |
| DF-HA: diclofenac etalhyaluronate  Responder rate is defined as the number (percentage) of subjects with at least 30% improvement from baseline in the numerical rating scale for pain. | | | | | |
