# Supplementary material for: Diclofenac–hyaluronate conjugate (diclofenac etalhyaluronate) intra-articular injection for hip, ankle, shoulder, and elbow osteoarthritis: a randomized controlled trial
Source: BMC Musculoskelet Disord. 2022 Apr 20;23:371. doi: 10.1186/s12891-022-05328-3 (PMC9022275; doi:10.1186/s12891-022-05328-3)
Supplement: Supplementary file 7 — Additional file 7: Supplementary Table 7. Change from baseline in acetaminophen consumption at each time point. [file 12891_2022_5328_MOESM7_ESM.docx]

**Additional file 7:** **Supplementary Table 7** Change from baseline in acetaminophen consumption at each time point.

| Joint | Week | Change from baseline (mg/day) | | | |
| --- | --- | --- | --- | --- | --- |
|  |  | DF-HA | | Placebo | |
|  |  | *n* | Mean ± SD | *n* | Mean ± SD |
| Hip | 1 | 46 | −178.1 ± 235.9 | 44 | −112.2 ± 226.8 |
|  | 2 | 46 | −115.9 ± 224.4 | 44 | −94.4 ± 214.6 |
|  | 4 | 46 | −115.5 ± 239.6 | 43 | −50.9 ± 243.0 |
|  | 6 | 46 | −108.3 ± 280.7 | 43 | −84.1 ± 230.3 |
|  | 8 | 46 | −91.8 ± 275.1 | 42 | −73.5 ± 215.4 |
|  | 10 | 45 | −151.5 ± 278.9 | 42 | −45.2 ± 203.6 |
|  | 12 | 44 | −119.7 ± 277.6 | 42 | −55.0 ± 224.7 |
| Ankle | 1 | 30 | −65.0 ± 150.4 | 30 | −99.4 ± 207.6 |
|  | 2 | 30 | −31.4 ± 137.3 | 30 | −110.0 ± 193.3 |
|  | 4 | 30 | 12.6 ± 196.3 | 30 | −71.3 ± 186.0 |
|  | 6 | 29 | 9.0 ± 243.7 | 30 | −57.0 ± 200.2 |
|  | 8 | 29 | −75.5 ± 155.1 | 30 | −95.7 ± 244.5 |
|  | 10 | 29 | −52.3 ± 166.0 | 29 | −98.5 ± 241.8 |
|  | 12 | 29 | −38.0 ± 175.7 | 28 | −55.6 ± 207.9 |
| Shoulder | 1 | 45 | −36.5 ± 166.1 | 45 | −18.1 ± 117.9 |
|  | 2 | 45 | −2.3 ± 225.6 | 45 | −36.6 ± 161.6 |
|  | 4 | 45 | 27.6 ± 164.5 | 44 | −0.3 ± 217.0 |
|  | 6 | 45 | 36.3 ± 163.1 | 44 | −23.0 ± 283.1 |
|  | 8 | 45 | −11.1 ± 238.3 | 44 | 35.5 ± 299.5 |
|  | 10 | 45 | −9.3 ± 250.3 | 44 | −26.4 ± 286.6 |
|  | 12 | 45 | −23.5 ± 239.9 | 44 | −31.2 ± 240.9 |
| Elbow | 1 | 25 | −89.0 ± 224.1 | 25 | −25.9 ± 87.7 |
|  | 2 | 25 | −2.3 ± 200.8 | 25 | −26.0 ± 114.7 |
|  | 4 | 25 | 53.2 ± 203.1 | 25 | 14.6 ± 158.0 |
|  | 6 | 25 | −9.2 ± 231.4 | 25 | −5.7 ± 166.3 |
|  | 8 | 25 | 43.5 ± 298.4 | 25 | 24.3 ± 189.8 |
|  | 10 | 25 | 18.3 ± 318.8 | 25 | 15.7 ± 187.6 |
|  | 12 | 25 | 15.8 ± 327.6 | 24 | 24.1 ± 170.6 |
| DF-HA: diclofenac etalhyaluronate; SD: standard deviation  Acetaminophen consumption was considered to be zero in subjects who did not take it. | | | | | |
