# Supplementary material for: Diclofenac–hyaluronate conjugate (diclofenac etalhyaluronate) intra-articular injection for hip, ankle, shoulder, and elbow osteoarthritis: a randomized controlled trial
Source: BMC Musculoskelet Disord. 2022 Apr 20;23:371. doi: 10.1186/s12891-022-05328-3 (PMC9022275; doi:10.1186/s12891-022-05328-3)
Supplement: Supplementary file 8 — Additional file 8: Supplementary Table 8. Change from baseline in range of motion at Week 12. [file 12891_2022_5328_MOESM8_ESM.docx]

**Additional file 8:** **Supplementary Table 8** Change from baseline in range of motion at Week 12.

| Joint |  | Change from baseline (deg) | |
| --- | --- | --- | --- |
|  |  | DF-HA | Placebo |
|  |  | Mean ± SD | Mean ± SD |
| Hip | *n* | 44 | 42 |
|  | Extension | 2.7 ± 5.3 | 0.4 ± 6.2 |
|  | Flexion | 4.2 ± 8.6 | 4.4 ± 12.9 |
|  | Internal rotation | 3.8 ± 9.7 | 4.6 ± 9.8 |
|  | Abduction | 0.3 ± 7.7 | 0.8 ± 5.7 |
| Ankle | *n* | 29 | 28 |
|  | Extension | 3.3 ± 5.0 | 3.0 ± 7.0 |
|  | Flexion | 6.2 ± 10.0 | 7.5 ± 13.6 |
| Shoulder | *n* | 45 | 44 |
|  | Forward flexion | 20.3 ± 27.2 | 11.7 ± 24.7 |
|  | Abduction | 18.2 ± 35.4 | 16.4 ± 27.6 |
|  | External rotation | 4.9 ± 16.3 | 8.0 ± 18.4 |
|  | Internal rotation | 2.0 ± 10.5 | 1.7 ± 14.6 |
| Elbow | *n* | 25 | 24 |
|  | Extension | 3.9 ± 6.3 | 0.4 ± 4.1 |
|  | Flexion | −0.1 ± 7.8 | 3.1 ± 9.6 |
|  | Pronation | 4.7 ± 9.3 | 3.8 ± 9.6 |
|  | Supination | 2.0 ± 5.2 | 4.2 ± 16.4 |
| DF-HA: diclofenac etalhyaluronate; SD: standard deviation | | | |
