# Supplementary material for: Diclofenac–hyaluronate conjugate (diclofenac etalhyaluronate) intra-articular injection for hip, ankle, shoulder, and elbow osteoarthritis: a randomized controlled trial
Source: BMC Musculoskelet Disord. 2022 Apr 20;23:371. doi: 10.1186/s12891-022-05328-3 (PMC9022275; doi:10.1186/s12891-022-05328-3)
Supplement: Supplementary file 9 — Additional file 9: Supplementary Table 9. Structural changes visible by X-ray imaging at the last assessment. [file 12891_2022_5328_MOESM9_ESM.docx]

**Additional file 9:** **Supplementary Table 9** Structural changes visible by X-ray imaging at the last assessment.

| Parameter | Hip | | Ankle | | Shoulder | | Elbow | | Total | |
| --- | --- | --- | --- | --- | --- | --- | --- | --- | --- | --- |
|  | DF-HA | Placebo | DF-HA | Placebo | DF-HA | Placebo | DF-HA | Placebo | DF-HA | Placebo |
| *n* | 46 | 44 | 30 | 30 | 45 | 45 | 25 | 25 | 146 | 144 |
| Osteophyte | 0 | 0 | 0 | 0 | 1 (2.2) | 2 (4.4) | 0 | 0 | 1 (0.7) | 2 (1.4) |
| Joint space narrowing | 5 (10.9) | 0 | 2 (6.7) | 1 (3.3) | 1 (2.2) | 2 (4.4) | 0 | 0 | 8 (5.5) | 3 (2.1) |
| Osteosclerosis | 1 (2.2) | 1 (2.3) | 1 (3.3) | 0 | 1 (2.2) | 0 | 1 (4.0) | 2 (8.0) | 4 (2.7) | 3 (2.1) |
| Epiphyseal deformity | 0 | 0 | 1 (3.3) | 0 | 0 | 1 (2.2) | 0 | 0 | 1 (0.7) | 1 (0.7) |
| DF-HA: diclofenac etalhyaluronate  Data are presented as *n* (%). | | | | | | | | | | |
