# Supplementary material for: Diclofenac–hyaluronate conjugate (diclofenac etalhyaluronate) intra-articular injection for hip, ankle, shoulder, and elbow osteoarthritis: a randomized controlled trial
Source: BMC Musculoskelet Disord. 2022 Apr 20;23:371. doi: 10.1186/s12891-022-05328-3 (PMC9022275; doi:10.1186/s12891-022-05328-3)
Supplement: Supplementary file 10 — Additional file 10: Supplementary Table 10. Symptoms of worsening during target joint examinations. [file 12891_2022_5328_MOESM10_ESM.docx]

**Additional file 10: Supplementary Table 10** Symptoms of worsening during target joint examinations.

| Target joint examination | Week | Hip | | | | Ankle | | | | Shoulder | | | | Elbow | | | | Total | | | |
| --- | --- | --- | --- | --- | --- | --- | --- | --- | --- | --- | --- | --- | --- | --- | --- | --- | --- | --- | --- | --- | --- |
|  |  | *n* | DF-HA | *n* | Placebo | *n* | DF-HA | *n* | Placebo | *n* | DF-HA | *n* | Placebo | *n* | DF-HA | *n* | Placebo | *n* | DF-HA | *n* | Placebo |
| Joint effusion | 1 | 46 | 0 | 44 | 0 | 30 | 1 (3.3) | 30 | 4 (13.3) | 45 | 1 (2.2) | 45 | 0 | 25 | 1 (4.0) | 25 | 0 | 146 | 3 (2.1) | 144 | 4 (2.8) |
|  | 2 | 46 | 0 | 44 | 0 | 30 | 0 | 30 | 3 (10.0) | 45 | 0 | 45 | 0 | 25 | 0 | 25 | 0 | 146 | 0 | 144 | 3 (2.1) |
|  | 4 | 46 | 0 | 43 | 0 | 30 | 0 | 30 | 1 (3.3) | 45 | 0 | 44 | 2 (4.5) | 25 | 0 | 25 | 0 | 146 | 0 | 142 | 3 (2.1) |
|  | 6 | 46 | 0 | 43 | 0 | 29 | 0 | 30 | 3 (10.0) | 45 | 0 | 44 | 1 (2.3) | 25 | 0 | 25 | 0 | 145 | 0 | 142 | 4 (2.8) |
|  | 8 | 45 | 0 | 42 | 0 | 29 | 0 | 30 | 3 (10.0) | 44 | 0 | 44 | 1 (2.3) | 25 | 1 (4.0) | 25 | 0 | 143 | 1 (0.7) | 141 | 4 (2.8) |
|  | 10 | 45 | 0 | 42 | 0 | 29 | 0 | 29 | 2 (6.9) | 44 | 0 | 44 | 1 (2.3) | 25 | 1 (4.0) | 25 | 0 | 143 | 1 (0.7) | 140 | 3 (2.1) |
|  | 12 | 44 | 0 | 42 | 0 | 29 | 0 | 28 | 1 (3.6) | 45 | 1 (2.2) | 44 | 1 (2.3) | 25 | 0 | 24 | 0 | 143 | 1 (0.7) | 138 | 2 (1.4) |
| Swelling | 1 | 46 | 0 | 44 | 0 | 30 | 5 (16.7) | 30 | 6 (20.0) | 45 | 0 | 45 | 1 (2.2) | 25 | 2 (8.0) | 25 | 1 (4.0) | 146 | 7 (4.8) | 144 | 8 (5.6) |
|  | 2 | 46 | 0 | 44 | 1 (2.3) | 30 | 4 (13.3) | 30 | 5 (16.7) | 45 | 1 (2.2) | 45 | 0 | 25 | 2 (8.0) | 25 | 0 | 146 | 7 (4.8) | 144 | 6 (4.2) |
|  | 4 | 46 | 0 | 43 | 0 | 30 | 3 (10.0) | 30 | 5 (16.7) | 45 | 0 | 44 | 1 (2.3) | 25 | 2 (8.0) | 25 | 0 | 146 | 5 (3.4) | 142 | 6 (4.2) |
|  | 6 | 46 | 0 | 43 | 0 | 29 | 3 (10.3) | 30 | 4 (13.3) | 45 | 2 (4.4) | 44 | 1 (2.3) | 25 | 2 (8.0) | 25 | 0 | 145 | 7 (4.8) | 142 | 5 (3.5) |
|  | 8 | 45 | 0 | 42 | 0 | 29 | 3 (10.3) | 30 | 5 (16.7) | 44 | 0 | 44 | 2 (4.5) | 25 | 2 (8.0) | 25 | 0 | 143 | 5 (3.5) | 141 | 7 (5.0) |
|  | 10 | 45 | 0 | 42 | 0 | 29 | 5 (17.2) | 29 | 6 (20.7) | 44 | 0 | 44 | 4 (9.1) | 25 | 2 (8.0) | 25 | 0 | 143 | 7 (4.9) | 140 | 10 (7.1) |
|  | 12 | 44 | 0 | 42 | 0 | 29 | 3 (10.3) | 28 | 4 (14.3) | 45 | 0 | 44 | 0 | 25 | 2 (8.0) | 24 | 0 | 143 | 5 (3.5) | 138 | 4 (2.9) |
| Redness | 1 | 46 | 0 | 44 | 0 | 30 | 0 | 30 | 0 | 45 | 0 | 45 | 0 | 25 | 0 | 25 | 0 | 146 | 0 | 144 | 0 |
|  | 2 | 46 | 0 | 44 | 0 | 30 | 1 (3.3) | 30 | 0 | 45 | 0 | 45 | 0 | 25 | 0 | 25 | 1 (4.0) | 146 | 1 (0.7) | 144 | 1 (0.7) |
|  | 4 | 46 | 0 | 43 | 0 | 30 | 0 | 30 | 0 | 45 | 0 | 44 | 0 | 25 | 0 | 25 | 0 | 146 | 0 | 142 | 0 |
|  | 6 | 46 | 0 | 43 | 0 | 29 | 1 (3.4) | 30 | 0 | 45 | 0 | 44 | 0 | 25 | 0 | 25 | 0 | 145 | 1 (0.7) | 142 | 0 |
|  | 8 | 45 | 0 | 42 | 0 | 29 | 0 | 30 | 0 | 44 | 0 | 44 | 0 | 25 | 1 (4.0) | 25 | 1 (4.0) | 143 | 1 (0.7) | 141 | 1 (0.7) |
|  | 10 | 45 | 0 | 42 | 0 | 29 | 0 | 29 | 0 | 44 | 0 | 44 | 1 (2.3) | 25 | 1 (4.0) | 25 | 0 | 143 | 1 (0.7) | 140 | 1 (0.7) |
|  | 12 | 44 | 0 | 42 | 0 | 29 | 0 | 28 | 0 | 45 | 0 | 44 | 1 (2.3) | 25 | 0 | 24 | 0 | 143 | 0 | 138 | 1 (0.7) |
| Warmth | 1 | 46 | 0 | 44 | 0 | 30 | 1 (3.3) | 30 | 3 (10.0) | 45 | 0 | 45 | 1 (2.2) | 25 | 1 (4.0) | 25 | 0 | 146 | 2 (1.4) | 144 | 4 (2.8) |
|  | 2 | 46 | 0 | 44 | 0 | 30 | 1 (3.3) | 30 | 2 (6.7) | 45 | 0 | 45 | 1 (2.2) | 25 | 0 | 25 | 1 (4.0) | 146 | 1 (0.7) | 144 | 4 (2.8) |
|  | 4 | 46 | 0 | 43 | 0 | 30 | 0 | 30 | 2 (6.7) | 45 | 0 | 44 | 3 (6.8) | 25 | 1 (4.0) | 25 | 0 | 146 | 1 (0.7) | 142 | 5 (3.5) |
|  | 6 | 46 | 0 | 43 | 0 | 29 | 0 | 30 | 3 (10.0) | 45 | 0 | 44 | 2 (4.5) | 25 | 1 (4.0) | 25 | 0 | 145 | 1 (0.7) | 142 | 5 (3.5) |
|  | 8 | 45 | 0 | 42 | 0 | 29 | 0 | 30 | 5 (16.7) | 44 | 0 | 44 | 0 | 25 | 1 (4.0) | 25 | 1 (4.0) | 143 | 1 (0.7) | 141 | 6 (4.3) |
|  | 10 | 45 | 0 | 42 | 0 | 29 | 0 | 29 | 3 (10.3) | 44 | 0 | 44 | 0 | 25 | 1 (4.0) | 25 | 0 | 143 | 1 (0.7) | 140 | 3 (2.1) |
|  | 12 | 44 | 0 | 42 | 0 | 29 | 0 | 28 | 4 (14.3) | 45 | 0 | 44 | 0 | 25 | 1 (4.0) | 24 | 0 | 143 | 1 (0.7) | 138 | 4 (2.9) |
| DF-HA: diclofenac etalhyaluronate  Data are presented as *n* (%).  Data indicate the subjects whose joint effusion/swelling/redness/warmth worsened (negative to borderline, negative to positive, borderline to positive) compared with baseline. | | | | | | | | | | | | | | | | | | | | | |
